# Requirements for a dashboard optimized for melanoma patient care through user-centered context exploration

**DOI:** 10.1038/s41598-024-67857-2

**Published:** 2024-07-29

**Authors:** Eva Maria Hartmann, Alisa Küper, Jessica Swoboda, Georg Christian Lodde, Elisabeth Livingstone, Catharina Lena Beckmann, Dirk Schadendorf, Sabine Sachweh

**Affiliations:** 1 Department of Computer Science, University of Applied Sciences and Arts Dortmund, 44227 Dortmund, Germany; 2 Department of Social Psychology: Media and Communication, University of Duisburg-Essen, 47057 Duisburg, Germany; 3 Institute for Artificial Intelligence in Medicine, University Hospital Essen, 45147 Essen, Germany; 4 Clinic for Dermatology, University Hospital Essen, 45147 Essen, Germany

**Keywords:** Melanoma, Dashboard, Requirements, User experience, Electronic health records, Context exploration, Health care, Skin cancer, Data integration, Software

## Abstract

For time-sensitive treatment of a patient with malignant melanoma, physicians must obtain a rapid overview of the patient’s status. This study aimed to analyze context-specific features and processes at the point of care to derive requirements for a dashboard granting more straightforward access to information. The Think-Aloud method, contextual inquiries, and interviews were performed with physicians from the Department of Dermatology at the University Hospital Essen in Germany. The user statements and observations that were obtained were grouped and categorized using an affinity diagram. Based on the derived subjects, requirements were defined, confirmed, and prioritized. The resulting affinity diagram revealed four topics of importance at the point of care. These topics are “Identifying and Processing the Important”, a comprehensive “Patient Record”, tasks and challenges in the “Clinical Routine”, and interactions and experiences with the available “Systems”. All aspects have been reflected in 135 requirements for developing context- and indication-specific patient dashboards. Our work has elucidated the most important aspects to consider when designing a dashboard that improves patient care by enabling physicians to focus on the relevant information. Furthermore, it has been demonstrated that the aspects most often mentioned are not context-specific and can be generalized to other medical contexts.

## Introduction

During the treatment of patients diagnosed with melanoma (black skin cancer), it is crucial for dermato-oncologists (dermatologists specialized in cancer) to obtain a complete understanding of the patient’s status immediately. Therefore, accessibility and timeliness of the relevant data are in focus. In recent years, various medical information systems have been used increasingly in the EU to support the process of treatment and documentation at the point of care^1^; however, the usability of many of these systems has been generally poor^2–7^. Usability is defined by the accuracy and completeness with which the physician can reach the desired goal (effectiveness), the effort that is needed for task completion (efficiency), and how enjoyable the interaction is throughout the process (enjoyment of use)^8^. Lack of usability can lead to errors that can be life-threatening to patients^3,9,10^ and numerous kinds of workarounds, where physicians use the information system differently, than it was intended, to reach their desired goal^10^. Without addressing the issue of optimizing software for the context in which it will be used, these problems will remain^11^.

To counteract this well-known problem, methodologies such as user experience^12^ that take the user, the setting, and the situation of a software use into the focus of the implementation have been introduced. Such a user-centered design can address the problem of high mental load, especially in situations in which many different types of information need to be gathered, combined, and compared under time pressure^13,14^. Early involvement of users in the development process and an initial, thorough context exploration can lead to the creation of systems with easier accessibility that support physicians in clinical routine and specific tasks^15,16^, thus increasing productivity^14^. However, this step is often skipped during the development of most systems^17^.

Prior research has also revealed common factors that influence the acceptance or rejection of medical information systems^2,11,18–20^. For the acceptance of a system, users have specified ease of use as a key factor. The ability of the system to integrate into a context-specific workflow and its helpfulness in achieving a set goal have also been labeled as essential. Privacy issues^20,21^, poor design, long system downtimes, and long response times have led medical staff to reject systems^5,19,20^.

Initiating software development by placing a focus on context exploration has proven to prevent developers from deriving (defining) inadequate and incomplete requirements^22,23^. Granting users influence on an application through participation in its development process^24^ leads to higher acceptance^25^ and can create enriched designs by including different user insights^26^. Such designs often focus especially on user-relevant factors and therefore reduce mental load and information loss^27^. By overcoming the challenges of time constraints in clinical routines and recruiting a sufficient number of participants to represent the context^28^, user-centered design can improve software acceptance if the identified key factors are interwoven into the solution^29^.

To exploit the advantages of a user-centered design, our approach involved a combination of methods from the field of user research to explore the context and workflows in which the software would be used. For this purpose, user statements and insights were gathered and combined to gain contextual knowledge. The derived requirements were phrased and ranked according to their impact on the user’s ability to achieve their objective. Following this approach, a complete set of requirements has been established based directly on observations of the context or user statements. These requirements, which form the basis of dashboard development, consider user-related factors, place a focus on data relevant to the treatment of melanoma patients, and offer contextual knowledge to enable workflow integration.

Our study aimed to define a reproducible starting point for context-sensitive dashboard development using early user involvement. Therefore, in this paper, we introduce a set of functional (FR) and non-functional requirements (NFR) for the context of melanoma treatment. Furthermore, we uncovered the underlying user’s intention related to the defined requirements by linking them with the important aspects of this context through an affinity diagram (AD). To address real-world problems in the context, we introduce the unique combination of using the insights gathered from the think-aloud method (TA) and contextual inquiry (CI) in an AD to define requirements.

## Methods

### Ethics statement

This study was approved by the Ethics Committee of the Medical Faculty (approval number 22-10814-BO for CI and survey) and the Faculty of Engineering (approval number 2111SPKA9493 for TA) of the University of Duisburg-Essen. All procedures were performed in accordance with the ethical standards of the institutional research committees, the 1964 Helsinki Declaration, and its later amendments or comparable ethical standards. All subjects provided their informed consent for inclusion on the TA in written form. The CI and survey protocols did not include any personal data.

### Approach

**Figure 1:**
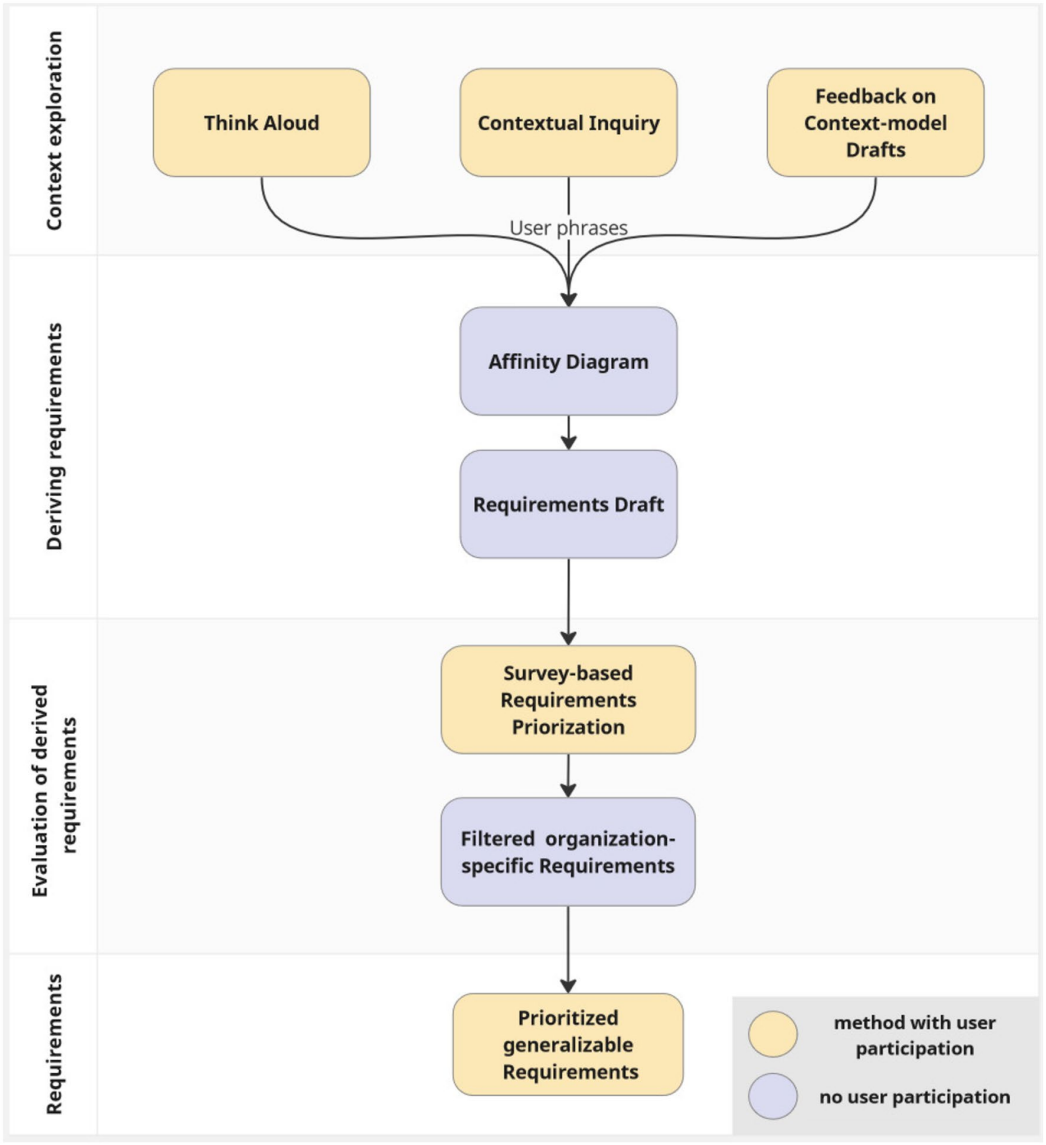
Pipeline derivation and evaluation requirements with a combination of qualitative user-centered methods.

For the definition of requirements, user phrases obtained by the TA method, CI, and continuous feedback regarding derived context descriptions were collected to serve as foundation of an AD revealing the topics most important to the context (Fig. 1). The requirements were then drafted and subsequently ranked regarding their priority by three experts. Physicians with various levels of expertise from the Department of Dermatology at the University Hospital Essen (UME) in Germany participated in these qualitative methods. Senior physicians, specialists, and residents who had just begun working in this department contributed their domain knowledge and personal input (Table 1). Finally, the requirements were reviewed with regard to their relationships to the hospital and excluded if not generally applicable.

**Table 1:** Methods used to derive and evaluate requirements in the context of melanoma patient treatment, their purposes in the information-processing pipeline, the numbers of participants, and the methods used to collect the data.

Method	Purpose in information processing	Focus of exploration	Number of participants	Data collection method
Think-Aloud	Context exploration	Preparation of tumor board	10	Screen recording, voice recording
Contextual inquiry		Ambulant treatment	2	Protocol
		Initial presentation	2	Protocol
Interviews		Drafts of context models	2	Protocol
Survey	Evaluation of derived requirements	Prioritization of derived requirements	2	Online survey

### Think-Aloud method

The TA^30,31^ method is common in user-centered approaches and has been used frequently to reveal problem areas and factors important to an inspected context^32^. It is conducted by asking participants to fulfill a task and at the same time express their thoughts verbally. These actions and thoughts are recorded and analyzed to depict the context and build designs based on this knowledge. This method is applicable to any scenario^33^, has been shown to be highly effective, and allows modeling and design based on voices of real users rather than external interpretations^33^.

The TA method was conducted with 10 participants from the UME in the field of melanoma treatment. The explored task was the preparation of a fictitious tumor board for a patient in a possibly metastatic setting. Task execution was captured via computer screen and voice recording. To simulate real-world circumstances, this process occurred in the clinic using real patient data from the hospital information systems. The time was limited to 15 min, and no interaction with the conductors occurred other than reminders to think aloud.

The TA provided deep insights into the interactions with the software, the relevant data, how they relate to one another, and how physicians translate the information into decisions. Nevertheless, the time required is very high, especially in this time-critical context. The setting and the task were as realistic as possible, and the participants were informed in advance that the study focused on their interaction with the software and not on their medical decisions. Despite this, some physicians were nervous, felt uncomfortable expressing their professional thoughts out loud, and maybe filtered their statements.

The data gathered was anonymized by voice alteration and transcription of the recorded voices. Afterwards, relevant phrases were extracted from the transcripts and used anonymously as part of the AD.

### Contextual inquiry

CI^16^ is a participatory method in user research, which involves the user in the development process and takes an entire context into focus. By accompanying physicians in conducting the tasks supported by software and observing their interactions, the nature of situations can be revealed. With this approach, direct insights into the setting, intents of usage, and utilization of available IT systems can be gathered^2^. This knowledge enables the design of software that uses context-specific wording and helps to identify standard solutions that might cause conflicts in this context. Even facts that are subconscious and difficult to articulate can be revealed using this method^17^. Revealing true needs is a strong basis for deriving requirements based on real insights rather than interpretation by external factors^28^. Conducting a CI is relevant for a holistic view of the context^15,34,35^.

CI was conducted with two participants each for the tasks of outpatient care and the initial presentation of new patients. The information gathered was documented in prepared protocols that focused on the setting, interruptions, and interactions with IT systems or colleagues. No personal information regarding the participating physicians was documented.

The CI gave an unfiltered view of the observed tasks, usual settings, and interactions with the medical staff and patients without impacting the physician’s time. The physicians were used to introducing new colleagues to their tasks and being accompanied, which made the CI very realistic and unvaried. Observations were only limited if the patient felt uncomfortable with an observer, or the observer did not want to accompany the medical treatment.

To facilitate empathy during the AD modeling process, user phrases were extracted from the protocols by rephrasing the insights in the first person. In addition, any questions that arose during the modeling phase were collected and discussed in semi-structured interviews with two dermato-oncologists. These insights were similarly extracted from the protocols and inserted into the AD.

### Affinity diagram

As part of contextual design, the AD^35^ was developed to reveal the topics important to an explored context. Building an AD begins with sorting user phrases and insights gained from qualitative methods according to their content. Obtaining a maximum number of statements per group leads to critical reflection, discovery of deeper insights, reorganization, and rephrasing of the groups. Afterwards, these groups are clustered into sub-topics, which again are grouped into topics. The specified groups, sub-topics, and topics are called affinities, from which the name of the diagram originates. During the modeling process, all groups, sub-topics, and topics are given descriptions in I-perspective, which supports empathy during the design process. This structuring of messages leads to the identification and clarification of areas of interest separated into manageable groups^36,37^ and serves as a solid foundation to derive user-founded requirements^38^.

### Requirements

In computer science, it is important to formulate a need that software must fulfill under given circumstances and known limitations^39^ in order to ensure the quality of the implementation and determine the purpose of the task. These needs and limitations are defined as requirements and exist in a hierarchical order with more specific sub-requirements. A distinction can be made between FRs, which define a functionality a software must provide, and NFRs, which describe characteristics a software must integrate. To differentiate their importance, a requirement can be prioritized into three levels. Functionalities or characteristics that are necessary to fulfill a task supported by the defined software have the highest priority and *shall* be implemented. Useful features that are not essential for task completion are requirements that *should* be included. Aspects facilitating work but not necessary for reaching the set goal *may* be supported by the software^40^.

Using these user-centered methods, we have derived a set of requirements for patient dashboard development and optimization in the context of melanoma treatment.

### Deriving requirements

To derive requirements based on qualitative data, the transcriptions of TA sessions and CI protocols were split into shorter phrases containing only one message. The user statements were then grouped into three hierarchies and labeled in I-perspective regarding their shared content by modeling the AD. Requirements were determined based on the resulting topics, sub-topics, and groups. Starting with the most abstract level (topics) of the AD, each level down to the very practical user statements was considered individually. The core message of the affinity was extracted and its influence on the dashboard was evaluated. In the next step, this insight was phrased as a new requirement or assigned to an existing one. Afterward, all requirements were examined to determine whether they described a functionality or a feature. Finally, the requirements were grouped by their focus in a hierarchical order, with more specific sub-requirements. The majority of requirements were derived from sub-topics and groups. In very few cases, general requirements were linked to single phrases or specialized sub-requirements referred to a topics.

### Evaluation of derived requirements

The derived requirements were evaluated for generalizability; those directly tied to the organization and its specific workflows were excluded. Furthermore, during a survey, the requirements were either prioritized or excluded to ensure correct interpretation of the user phrases by the derived requirements. In this process, two physicians and an IT specialist rated the requirements regarding their prioritization. The following four options were available to classify the requirements, the corresponding values of which are given in brackets: Functionalities or characteristics necessary to fulfill a task supported by the defined software have the highest priority and *shall* (3) be implemented. Useful features that are not essential for task completion are requirements that *should* (2) be included. Aspects facilitating work but not necessary for reaching the set goal *may* (1) be supported by the software. Participants could also mark a requirement as not necessary (0) to check for or add missing aspects. After adding the priorities, the arithmetic mean of the prioritization was finally applied to the list of requirements.

Overall, this study combined the TA method and CI to collect user-statements during the work process. These statements were subsequently sorted in an AD regarding their shared topics. These topics and statements were used to derive requirements that were directly related to the context. To prevent misinterpretation of these statements within the requirements, they were finally ranked regarding their prioritization or filtered out by conducting a survey.

## Results

### Affinity diagram

Overall, 507 user phrases were extracted from the qualitative data (text from transcripts and protocols) and combined in an AD. The resulting topics were then used to define 135 requirements. Considering the results side by side, it revealed not only required functionalities and features but also underlying user intentions.

**Figure 2:**
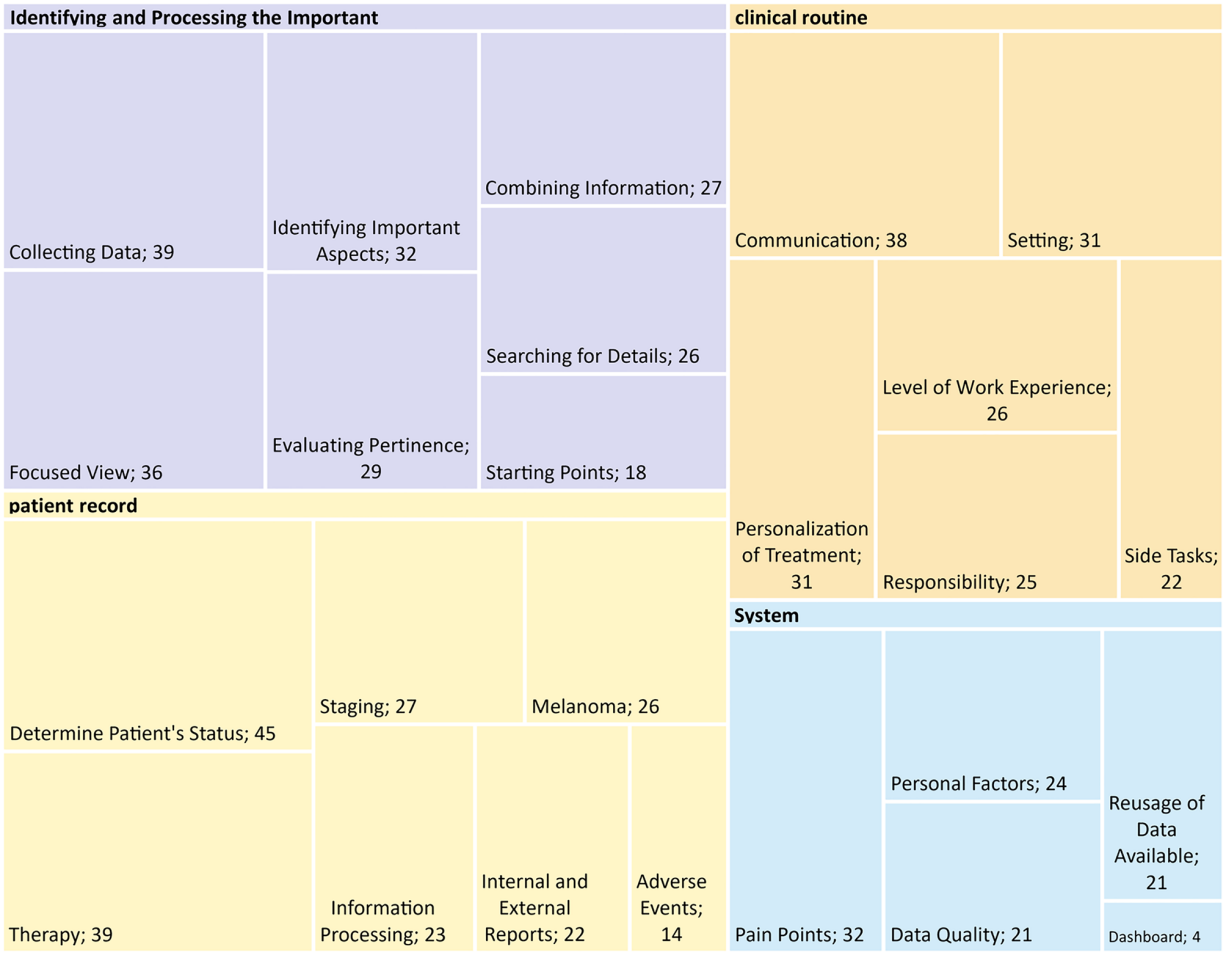
Four main topics (colored areas) and sub-topics of the affinity diagram. The boxes containing the sub-topics have been scaled based on the number of underlying groups.

Analysis of 507 user phrases organized in an AD revealed four main topics relevant to the context (Fig. 2). These topics included identification of important data related to treatment (“Identifying and Processing the Important”), the importance of a comprehensive patient record (“Patient Record”), integration with clinical routine and experience (“Clinical Routine”), and interaction with available systems (“Systems”). The following section only focuses on AD’s first top hierarchies. The complete set of affinities is included in the supplement (excel sheet tab: “1-overview-affinities”).

Containing 214 sub-elements, “Identifying and Processing the Important” was the most-often mentioned aspect of the task of treating melanoma patients. Outlined by eight sub-topics, it encompasses the challenges of finding the proper *starting point*, *collecting data,* and *searching for details* important to the case even under *time pressure*. All the information gathered must be evaluated for pertinence (behavior over time) and combined with data from other sources. While maintaining a focused view of the current case, the disease’s progression must also be considered.

A comprehensive “Patient Record” displaying all the required information was the second-most frequently referenced aspect. Information that must be included in a melanoma-specific dashboard was identified by 203 sub-elements in six sub-topics: *staging*, *adverse events*, *internal and external reports*, *therapies, determining the patient’s status*, and details on the *melanoma* itself.

The subject of “Clinical Routine” was mentioned in 179 sub-elements. The six sub-topics consider the *setting* and the *level of work experience* of the physicians. Having the patient in focus reflects the *responsibility* of physicians towards their patients, the aim of *personalizing treatment*, and *communication* between both physicians and colleagues and physicians and patients. Furthermore, the importance of side tasks during treatment was emphasized.

The last main topic relates to the “Systems” available and interactions with them. In five sub-topics containing 107 sub-elements, *pain points (*pitfalls) in software interaction, the influence of *personal preferences and habits*, *data quality*, and the possibilities of *reusing data* already known by the system(s) were in focus. This topic also includes references to the dashboard as a new system.

### Requirements

A total of 426 of 507 user phrases were reflected in 135 requirements for a dashboard supporting the treatment of melanoma patients, whereas 81 user statements could not be used to define requirements for a dashboard, and three requirements were excluded due to their strong relationship to the workflow at the hospital itself.

The 135 derived requirements could be further classified into 97 which defined functional aspects, and 38 which referred to non-functional characteristics that must be considered. Through prioritization, 39 of the 97 functionalities were marked as indispensable (*shall*) for the treatment of melanoma patients. To facilitate the process, 52 more of the 97 requirements should be implemented, and six may be an opportunity to simplify the workflow and information retrieval further. The functionalities were grouped into 13 main requirements, which are shown in Table 2 in conjunction with the affinities from which they were derived. In the following description, only the top hierarchy level of requirements with the most frequent assignments to affinities has been presented in detail. The complete catalog of requirements and sub-requirements has been included in the supplement (excel sheet tab: “2-overview-requirements”).

**Table 2:** The 13 main FRs and five main NFRs and their relationships to the user statements and topics in the affinity diagram.

Main requirement ID	Requirement “The dashboard shall/ should/may include…”	Count affinity per requirement group	Affinity-1st sub-topics
FR00	An all-encompassing view of all a patient’s data	69	Determine patient’s status, usage of data available, responsibility, side tasks, personalization of medicine, level of work experience, data collection, identifying important aspects, combining information, searching for details, focused view
FR01	The current staging	22	Staging
FR02	Ensuring access to external and internal findings	51	Internal and external reports, responsibility, side tasks
FR03	The master data of the patient	15	Data quality
FR04	The latest tumor board protocols	12	Determine patient’s status, starting points
FR05	The medication	6	Determine patient’s status, side tasks
FR06	The current laboratory values	16	Determine patient’s status
FR07	Progress documentation	29	Determine patient’s status
FR08	The histology of the tumor	9	Determine patient’s status
FR09	An overview of all melanoma-specific aspects	45	Melanoma
FR10	The therapy	42	Therapy
FR11	The reference to external documents needed for the fulfillment of the tasks	24	Communication, level of work experience
FR12	Searching in and filtering lists	55	Searching for details, treatment factor time
NFR00	Enable the user to get an overview even when time is short	111	Information processing
NFR01	Meet the requirements for data protection	10	Data quality
NFR02	Limit the respective view to the use case‐dependent essential information	29	Focused view
NFR03	Arrange elements based on the logic of melanoma treatment	15	Focused view
NFR04	Optimize work in any task‐appropriate usage setting	24	Personal factors, communication

Requirements have been represented by their ID and their specific requirement text. The affinities have been reflected in the counts of affinities associated with the requirement group (main requirement with sub-requirements), alongside assigned first sub-topics.

Based on the number of user phrases associated with the functional aspects, four key factors could be identified. The first factor (*FR00),* associated with 69 user phrases, states that an all-encompassing view of all a patient’s data, with straightforward access, is the most important functionality. A second consideration is the importance of sorting and filtering functionalities to search available data for necessary information and to reflect the patient’s status in chronological order. This aspect was mentioned in 55 user statements (*FR12).* Mentioned by 51 users, access to original external and internal reports ranked third. These reports are main sources of information and are useful for identifying information regarding documentation of progress and fulfillment of side tasks related to patients (*FR02*). The fourth aspect, mentioned 45 times by participants, refers to obtaining an overview of the melanoma itself (*FR09*). This overview includes the details of the primary tumor, in particular its location, tumor diagnosis, classification, lymph node status, location and confirmation of metastases, and the development of the tumor over time (Table 3).

**Table 3:** Melanoma-specific sub-requirements, represented by ID, priority, and a specific requirement text.

Requirement ID	Priority	Functionality	Count statement per requirement
FR09.01	SHALL	The current status of the primary tumor	5
FR09.01.01	SHALL	The localization of the primary tumor	1
FR09.02	SHALL	The tumor diagnosis	7
FR09.02.01	SHOULD	The confirmation status of the diagnosis	4
FR09.03	SHALL	The TNM classification	7
FR09.03.01	SHALL	The tumor stage	3
FR09.04	SHOULD	The change of the tumor stage over time	8
FR09.05	SHOULD	The current status of the lymph nodes	4
FR09.06	SHALL	Information regarding possible metastases	2
FR09.06.01	SHOULD	The type and location of the metastasis	1
FR09.06.02	SHOULD	The status of the confirmation of metastasis findings	2

The user phrases have been reflected in the counts of statements associated with each requirement.

Apart from the functionalities required, 38 NFRs were also derived to describe the characteristics that a dashboard should exhibit. According to the prioritization by two domain experts and one IT-expert, 18 of these attributes are indispensable (*shall*), 18 could simplify (*should*), and two *may* enrich the process of melanoma patients’ treatment. The characteristics have been grouped into five main requirements, along with their associations with the affinities from which they were derived.

Mentioned most often, with 111 assigned affinities, is the requirement (*NFR00*) for support of information processing through an overview that not only contains all necessary information but also is especially easy to interpret. Assigned to 29 user phrases, a view adapted to the task at hand can assist in obtaining a focused view of the relevant information (*NFR02*). The adaptation of the software to different settings was mentioned in 24 affinities (*NFR04*). Such adaptation includes flexibility with personal preferences and habits and support for communication. Optimizing a view while considering melanoma domain knowledge can provide experts with a focused and more easily interpretable view (*NFR03*), as highlighted by 15 user statements. According to 10 affinities, a dashboard must also ensure data protection, quality, and reproduction true to the original information (*NFR01*).

## Discussion

Our work has provided a set of important requirements at the point of care in the treatment of melanoma patients. These requirements were derived from user phrases using an AD to identify and clarify the most important aspects. Using CI and the TA method, insights were gained by observing the working routine rather than making assumptions.

In contrast to other publications that have employed user-centered approaches only for final evaluation, this approach emphasized the value of early involvement and thorough context exploration before designing and implementing the system. Although the benefits of direct insights into context have been shown previously, user-centered methods have remained sparsely used in the field of medicine, especially at the initial stages of the development process. For example, CI has been used to gain insights into the workflows of telecardiology and derive requirements based on these^41^. The AD has been used as a basis for determining topics that need to be investigated in further detail for daily morning surgical rounds in intensive care units (ICUs)^42^. Another publication has described two studies that applied a combination of CI and AD to derive requirements for the dictation process of physicians and a nursing documentation system^43^. By bringing these approaches to the field of melanoma treatment, unique requirements for this context were derived in the present study. To our knowledge, no other work has combined the TA, CI, and AD in one study to derive requirements for the development of a patient dashboard.

The results reported here demonstrate the strength of this approach. They have highlighted generalizable aspects of dashboard development and have also provided detailed insights into domain-specific needs. The factors mentioned most often refer to general aspects, such as gaining a comprehensive view even under time pressure, which would be transferable to other dashboard projects. Additionally, detailed insights into the medical aspects of melanoma have been highlighted. This includes specific prioritized data and a comprehensive view of the patient’s information that is needed due to the high impact of cancer on the patient’s entire body. Different types of cancer have different markers and values, such as typical genetic alterations, which need to be considered in diagnosis and treatment. However, similar diagnostic procedures are often used, resulting in the same data types. This implies that the very context-specific requirements are also highly reusable for other types of cancer. Further research will have to evaluate the generalizability of information-related requirements as well as those referring to general aspects. Addressing the related contexts of (skin) cancer treatment will give first valuable insights into the scalability of the requirements.

One limitation of this approach is that each context must be investigated separately to obtain context-specific insights. Context exploration must be performed for each discipline and does not offer a generally valid solution for every application. Different contexts might involve different settings the software will be used in, like mobile usage or limited software interaction possibilities as usual in operation rooms. Different diseases will lead to different data being prioritized during decision-making. However, the generalizable aspects identified can be used to focus primarily on domain-specific details in future projects. Requirements referring to getting an overview of the patient’s status or searching and filtering functions are most likely to be transferable even to other disciplines. More specialized aspects, like the information prioritized, might be partly reusable for other cancers and highly reusable for other skin cancers. Those differences will always need to be evaluated in different settings.

A further limitation is that these results were derived based on a single hospital. Whether our requirements can be implemented directly in other hospitals needs to be evaluated. Differences in workflows and related data acquisition might as well be hindering the transferability of the requirements, as might differences in the user group regarding the working experience and the user’s technical affinity. Those aspects might lead to different data needed during the treatment and shift focus on their prioritization. As treatment for melanoma follows guidelines, the transferability of the requirements might strongly depend on the implementation of the guidelines in different hospitals.

Overall, the applicability of the TA method and especially CI have proven to be valuable in this time-critical context. Combining, on the one hand, the TA, which has a specific task in a staged setting in focus, with the CI, where physicians are accompanied during working routines, has opened a broad view of the context from several directions. They have demonstrated utility in gaining deep insights into this complex context, making it accessible even for people not directly involved in that context, such as developers, through an AD.

The derived requirements can serve as a foundation for iteratively incorporating these needs into a dashboard design, allowing the developer to reflect on the design with the user’s intentions in mind. Deriving requirements with a user-centered approach was only the first step in the development of a context-, patient- and user-optimized dashboard for the treatment of melanoma. Future research needs to explore how the relevant data can be termed, grouped^44,45^, and visualized^45,46^ while addressing different, even contradictory personal preferences^47,48^. Data coming from different clinical information systems might be, duplicated, incomplete, and contradictory. The challenge of preventing the user from overload while evaluating the combined information will need to be addressed^49^.

Varying software interfaces of the different information systems can cause problems in the merging of data. It needs to be explored how interoperability between these systems can be established while ensuring data quality^20,49^. Combining this research with continuous user feedback and testing for developed concepts, prototypes, and the end product, can ensure the correct implementation^15^ and the usability of medical systems can be sustainably improved.

In summary, this study made two main contributions to the field of medical informatics. On one hand, it identified critical requirements for melanoma patient treatment at the point of care through user-centered methods. Through early user involvement and thorough context exploration, our approach provided valuable insights into workflows and specific needs. The highlighted generalizable aspects can, furthermore, be used as a foundation for dashboards in different medical domains. On the other hand, the study demonstrated the utility of CI and TA in understanding complex contexts, making the knowledge gained accessible to developers through an AD and reusable by the definition of requirements.

It points out a combination of methods that are proven to be applicable in the time-critical and complex context of medical treatment to define an implementation instruction for a dashboard that is aware of the patient’s context and the user. This user-centered focus in development ensures that the dashboard will address real-world needs and counteract errors related to missing context integration. Thereby, making the access to data easier for the physicians allows them to focus only on the important aspects and make decisions based on them.

### Supplementary Information

Supplementary Information.

## Data Availability

The authors confirm that the data supporting the findings of this study is available within the article and its supplementary materials or upon reasonable request to the corresponding author.
